# Investigation on an ammonia supply system for flue gas denitrification of low-speed marine diesel

**DOI:** 10.1098/rsos.171469

**Published:** 2017-12-20

**Authors:** Xiankun Huang, Han Yuan, Jian Zhao, Ning Mei

**Affiliations:** Marine Engineering Department, Ocean University of China, 238 Songling Road, Qingdao, 266100, People's Republic of China

**Keywords:** denitrification, ammonium bicarbonate, heat transfer coefficient, SCR, low-speed marine diesel

## Abstract

Low-speed marine diesel flue gas denitrification is in great demand in the ship transport industry. This research proposes an ammonia supply system which can be used for flue gas denitrification of low-speed marine diesel. In this proposed ammonia supply system, ammonium bicarbonate is selected as the ammonia carrier to produce ammonia and carbon dioxide by thermal decomposition. The diesel engine exhaust heat is used as the heating source for ammonium bicarbonate decomposition and ammonia gas desorption. As the ammonium bicarbonate decomposition is critical to the proper operation of this system, effects have been observed to reveal the performance of the thermal decomposition chamber in this paper. A visualization experiment for determination of the single-tube heat transfer coefficient and simulation of flow and heat transfer in two structures is conducted; the decomposition of ammonium bicarbonate is simulated by ASPEN PLUS. The results show that the single-tube heat transfer coefficient is 1052 W m^2^ °C^−1^; the thermal decomposition chamber fork-type structure gets a higher heat transfer compared with the row-type. With regard to the simulation of ammonium bicarbonate thermal decomposition, the ammonia production is significantly affected by the reaction temperature and the mass flow rate of the ammonium bicarbonate input.

## 1. Introduction

The marine diesel engine is competitive in large ships because of its high fuel efficiency and power output. However, the removal of NO*_x_* from diesel engine exhaust is a great challenge in environmental catalysis and air pollution control [1–5]. At present, the denitrification technologies include selective non-catalytic technology (SNCR) and selective catalytic technology (SCR). Both SNCR and SCR (table 1) techniques use chemical reducing agents to produce ammonia and react with the NO*_x_* in flue gas. For the SNCR technology, the reducing agent is directly sprayed into the diesel flue gas, with the denitrification reaction temperature at 900–1100°C. For the SCR technology the reaction temperature is lower; under the action of the catalyst, the reducing agent reacts with NO_*x*_ in the temperature range between 300°C and 400°C [6–9]. In this paper, the emphasis mainly focuses on the flue gas denitrification of low-speed marine diesel.

**Table 1.** Nomenclature.

*A*	heat transfer area of heat exchanger (m^2^)	*greek letters*	
*c*_p_	specific heat capacity of the cold fluid (kJ/(kg °C))	α	heat transfer coefficient (kW/(m K))
*E*_1_	heat output (kW s^−1^)	δ	thermal conductor thickness (mm)
*E*_2_	heat supplied by the heat transfer oil (kW s^−1^)	$\bar{\lambda }$	thermal conductivity of materials used in heat conducting parts (kW/(m K))
*e*	thermal enthalpy of heat transfer (kJ kg^−1^ °C)	ρ	density of cold fluid (kg m^−3^)
*H*	effective working area (m^2^)		
*Q*	heat transfer (kJ h^−1^)		
Q	quality of the pump oil (kg h^−1^)		
*q*_v_	volume flow of cold fluid (m^3^ h^−1^)		
*t*_1_	oil temperature flow out of the flue		
*t*_2_	oil temperature flow into the flue		
*t*_b1_	oil temperature at the heat exchangers’ inlet (°C)		
*t*_b2_	oil temperature at the heat exchangers’ outlet (°C)		
*t*_c1_	inlet temperature of the cold fluid (°C)		
*t*_c2_	outlet temperature of the cold fluid (°C)		
$\Delta t_{m}$	logarithmic mean temperature (°C)		
$\Delta t_{\max}$	heat exchange surface temperature difference of the larger side (°C)		
$\Delta t_{\min}$	heat exchange surface temperature difference of the lower side (°C)		

The reducing agent selection for the SCR reaction is based on its physical properties, economy and safety. Generally, three kinds of reducing agents, which contain ammonia, are commonly used in power plants for flue gas denitrification: ammonia, ammonia–water mixture with a concentration at 20–30% [10] and urea [11–12]. However, their application on shipboard is quite a different matter due to the safety concern on the transportation and storage of reducing agents, as well as requirement of the limited bulk size of the ammonia supply system. The ammonia and high-concentration ammonia–water mixture are deemed not suitable for safety reasons [13], while the urea-based system is much more complex, which leads to an unacceptable exorbitant initial investment [14].

It is important to note that compared with above-mentioned reducing agents, ammonium bicarbonate shows distinct advantages for its safety storage, cheaper price and lower thermal decomposition reaction temperature, which make it a promising reducing agent. The only problem is the production of a harmful side product, carbon dioxide, during the decomposition of ammonium bicarbonate. As the efficiency of the denitrification reaction depends much on the ammonia volume fraction in the flue gas, the produced carbon dioxide will dilute the ammonia volume fraction [15], which ultimately leads to a lower denitrification reaction efficiency. Therefore, in the ammonium bicarbonate-based ammonia supply system, the carbon dioxide should be separated from the products mixture.

In addition, the thermal decomposition chamber [16] is a key device of the ammonia supply system. Based on the structure of the tube bundle, the heat exchanger can be classified as the plate type or the shell-tube type [17–20]. Compared with the plate type, the shell-tube heat exchanger has higher heat exchange effects. The arrangement of tubes has a great influence on the heat transfer effect of the heat exchanger. Therefore, experiments on the measurement of the heat transfer coefficient and simulation for the thermal decomposition chamber were conducted.

In this paper, an ammonia supply system was proposed for flue gas denitrification. The ammonium bicarbonate is used as the ammonia carrier in this system. The heat exchangers of the thermal decomposition chamber and the ammonia evaporator play important roles in the ammonia supply system, thus emphasis was laid on the thermodynamic performance of the heat exchanger. Besides, visualization experiments on measurement of the heat transfer coefficient and simulation for the thermal decomposition chamber were conducted. Moreover, the thermal decomposition of ammonium bicarbonate in the ammonia supply system was simulated by ASPEN PLUS; the effects of ammonium bicarbonate concentration and the reaction temperature of thermal decomposition on the ammonia production were analysed.

## 2. Fundamentals of ammonia supply system for flue gas denitrification

**Figure 1.**
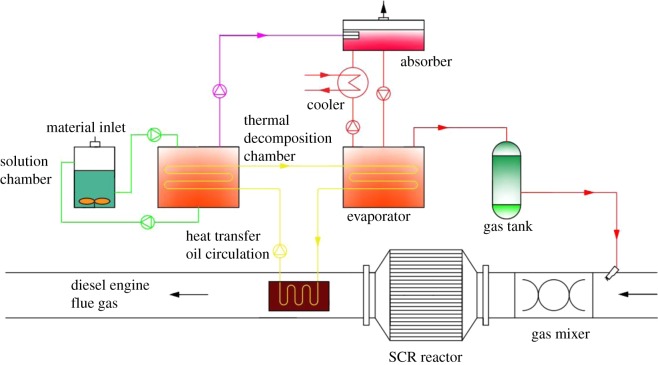
Schematic of an ammonia supply system for flue gas denitrification of low-speed marine diesel.

Figure 1 is the schematic of an ammonia supply system for flue gas denitrification of low-speed marine diesel.

The ammonia supply system mainly consists of the following components:

— heat transfer oil circulation system,
— ammonium bicarbonate thermal decomposition system,
— system for separation of carbon dioxide, and
— catalytic reduction step of ammonia with NO*_x_*.

In the heat transfer oil circulation system, oil is first heated by the exhaust heat of flue gas and then pumped into the ammonium bicarbonate thermal decomposition chamber and the ammonia evaporator. The output of the heat exchanger is calculated as follows [21]:

2.1 $$E_{1}=\frac{H(t_{1}-t_{2})}{\delta }\lambda .$$

The heat supplied by the hot oil is calculated as follows:

2.2 $$E_{2}=q(t_{b1}-t_{b2})e.$$

In the ammonium bicarbonate thermal decomposition system, ammonium bicarbonate is dissolved in the ammonium bicarbonate solution chamber and then pumped into the ammonium bicarbonate thermal decomposition chamber. The ammonium bicarbonate is decomposed by absorption of heat from the hot oil, and the gases carbon dioxide, ammonia and some water vapour are produced by the ammonium bicarbonate thermal decomposition. The chemical reaction is shown as follows:

2.3 $$\text{N}{\text{H}}_{4}\text{HC}{\text{O}}_{3}(\text{aq})\to \text{N}{\text{H}}_{3}(\text{aq})+\text{C}{\text{O}}_{2}(\text{g})+{\text{H}}_{2}\text{O},\;\Delta H=64.87\;\text{kJ}\;\text{mo}{\text{l}}^{-1}.$$

After thermal decomposition, dilute solution is pumped into the solution chamber to continue dissolving ammonium bicarbonate.

In the process of separating the carbon dioxide, the gases produced by the thermal decomposition chamber are pumped into the absorber. Owing to the high reaction rate of the ammonia with water, ammonia vapours are all dissolved rapidly, and only a small amount of carbon dioxide will be dissolved, with most of the carbon dioxide discharged from the absorber. Therefore, carbon dioxide can be separated from the mixture of gases. Because of the fully dissolved ammonia gas, the concentrated ammonia solution in the absorber is pumped into the evaporator. Ammonia gas is desorbed by the absorbed heat from the hot oil in the evaporator. After evaporation, dilute ammonia solution is pumped into the cooler to be cooled, and then pumped into the absorber to absorb ammonia gas. Ammonia gas desorbed in the absorber is pumped into the ammonia gas tank. Thereby, carbon dioxide is separated by the system between absorber, the evaporator and the cooler. The relevant chemical reactions are as follows:

Ammonia gas absorption:

2.4 $$\text{N}{\text{H}}_{3}(g)+{\text{H}}_{2}\text{O}(\text{l})\to \text{N}{\text{H}}_{4}\text{OH(aq)},\;\Delta H=-34.748\;\text{kJ}\;\text{mo}{\text{l}}^{-1}.$$

Ammonia gas desorption:

2.5 $$\text{N}{\text{H}}_{4}\text{OH(aq) }\to \text{N}{\text{H}}_{3}(\text{g})+{\text{H}}_{2}\text{O(l) , }\;\Delta H=34.748\;\text{kJ}\;\text{mo}{\text{l}}^{-1}.$$

In the catalytic reduction step of ammonia with NO*_x_*, the ammonia in the gas tank is injected into the diesel flue gas through the nozzle. The ammonia and flue gas are mixed with a mixer; and the mixture enters the SCR reactor. Under the action of the catalyst, ammonia reacts with NO*_x_* to produce a harmless gas N_2_. The relevant chemical reactions are as follows:

2.6 $$\text{4N}{\text{H}}_{3}(\text{g})+4\text{NO(g) }+{\text{O}}_{2}(\text{g})\to 4{\text{N}}_{2}(\text{g})+6{\text{H}}_{2}\text{O(g) },\;\Delta H=-1627.2\;\text{kJ}\;\text{mo}{\text{l}}^{-1}.$$

2.7 $$\text{4N}{\text{H}}_{3}(\text{g})+\text{2N}{\text{O}}_{2}(\text{g})+{\text{O}}_{2}(\text{g})\to 3{\text{N}}_{2}(\text{g})+6{\text{H}}_{2}\text{O(g) , }\;\Delta H=-\text{1309}.\text{17}\;\text{kJ}\;\text{mo}{\text{l}}^{-1}.$$

The working condition of the ammonia supply system can be calculated by the reaction equations listed in this section.

## 3. Comparison and initial calculation

### 3.1. Comparison of ammonium bicarbonate with urea

The traditional material urea, which is used in traditional denitrification, has high ammonia content and less carbon dioxide in its product. The urea solution is injected into the flue gas channel, using flue gas heat decomposition of ammonia, which application mode is used in the denitrification reaction. The new system proposed in this paper is for low-speed marine diesel engines, and the temperature of its flue gas is low enough to decompose urea, so the ammonia storage material selects ammonium bicarbonate instead of urea. But there is more carbon dioxide in the pyrolysis products of ammonium bicarbonate, and the pyrolysis products that enter directly into the flue gas pipeline affect the efficiency of the denitrification reaction. So the carbon dioxide separation step of the ammonium bicarbonate thermal decomposition is carried out before injection into the flue gas channel (table 2).

**Table 2.** Comparison of two reducing agents.

		Moore quality	unit molar ammonia	decomposition temperature	NO*_x_* conversion efficiency at different temperatures [22]		
reducing agent	chemical formula	g mol^−1^	mol mol^−1^	°C	200°C	300°C	400°C
urea	(NH)_2_CO	60.07	2	140	18	55	90
ammonium bicarbonate	NH_4_HCO_3_	79.06	1	60	22	65	90

The new system process proposed in this paper is different from the traditional denitrification system using urea, because the thermal decomposition temperatures of ammonium bicarbonate and urea do not overlap. In addition, in ammonia production, the thermal decomposition enthalpy of ammonium bicarbonate is lower than that of urea.

The relevant chemical reactions are as follows:

3.1 $$\text{N}{\text{H}}_{4}\text{HC}{\text{O}}_{3}(\text{aq})\to \text{N}{\text{H}}_{3}(\text{aq})+\text{C}{\text{O}}_{2}(\text{g})+{\text{H}}_{2}\text{O, }\;\Delta H=\text{64}.\text{87}\;\text{kJ}\;\text{mo}{\text{l}}^{-1}.$$

3.2 $$\text{CO(N}{\text{H}}_{2}\text{) (aq) }+{\text{H}}_{2}\text{O(l) }\to 2\text{N}{\text{H}}_{3}(\text{aq})+\text{C}{\text{O}}_{2}(\text{g}),\;\Delta H=\text{161}.\text{5}\;\text{kJ}\;\text{mo}{\text{l}}^{-1}.$$

NO*_x_* control is the most important issue in marine diesel engine emissions control, especially in offshore ports. When ships are sailing, the diesel engine operates in low operating conditions, so that the exhaust gas temperature is lower than the normal operating temperature. In low operating conditions, the temperature required for thermal decomposition of ammonium bicarbonate is more suitable than that for urea.

This paper is intended to describe this new system and describes the working of the system design.

### 3.2. Calculation results based on a certain type of marine diesel engine

The amount of ammonia required for the SCR reactor depends on the amount of NO*_x_* in the flue gas emissions of the diesel engine. This paper chooses the emission data of a certain type of marine diesel engine under different working conditions. It is intended to meet IMO Tier3 requirements by installing an SCR system. The following technical requirements for the SCR catalyst should be calculated. Table 3 presents the denitrification process of the material which is calculated.

**Table 3.** The working condition of the ammonia supply system as the workload of the marine engine varies.

workload of the marine engine	power (kW)	NO*_x_* emissions (g kWh^−1^)	exhaust flow (m^3^ h^−1^)	NO*_x_* (g s^−1^)	NH_3_ (g s^−1^)	NH_4_HCO_3_ (g s^−1^)	*E*_1_ (kW s^−1^)	*E*_2_ (kW s^−1^)
100%	1760	10.00	10800	4.89	2.85	13.27	10.90	13.07
75%	1320	10.39	8721	3.81	2.22	10.34	8.49	10.19
50%	880	8.33	6123	2.04	1.89	5.53	4.54	5.45
25%	440	8.43	4469	1.03	0.60	2.80	2.30	2.75
10%	176	10.51	3580	0.51	0.30	1.39	1.14	1.37

Based on typical workload of the marine engine, the working condition of the ammonia supply system is calculated and the results are given in table 3. According to the different operating conditions of the marine engine, we calculate the amount of NO*_x_* in the flue gas, so as to obtain the ammonia operating system parameters.

Then ammonium bicarbonate decomposition is critical to the proper operation of the ammonia supply system, and hence much work has been done on the thermal decomposition chamber in this paper. In this study, both experimental study and simulation were conducted to investigate the thermal decomposition characteristic of ammonium bicarbonate solution.

## 4. Experiment and simulation for the ammonia supply system

### 4.1. Single-tube heat transfer coefficient measured by experiments

The heat transfer coefficient of the ammonium bicarbonate thermal decomposition chamber is the key parameter which dominates system performance. In this study, a test bench was established to conduct the experimental investigation. The experimental test bench is mainly composed of heater, tank, pump, flowmeter, valve, thermocouple and switch. Hot fluid passes through a pipe with a size of *φ*20, and cold fluid through the acrylic tube with a size of *φ*50. The length of the heat transfer section is 500 mm. The outer surface of the heat exchanger is covered with an insulation layer, which can reduce the heat loss. To observe experimental phenomena, a window is opened in the insulation layer.

**Figure 2.**
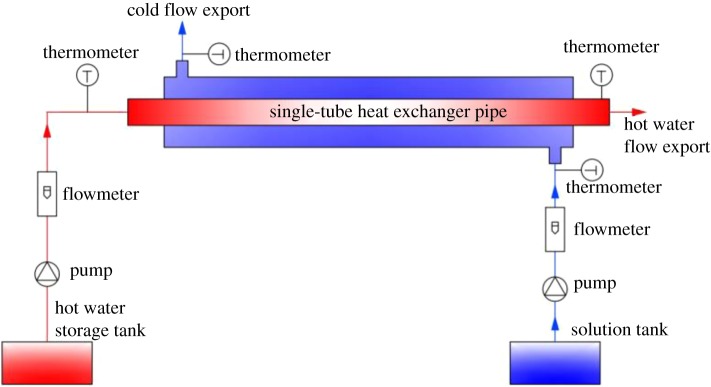
Schematic diagram of single-tube heat transfer test device.

The experimental procedure was as follows:

The experimental device was connected according to the design shown in figure 2. The heat transfer coefficient of the tube is measured by experiment. The experiment is heat transfer by the process of convection between a hot and cold fluid in the casing of the heat exchanger. According to the dissolution characteristics of ammonium bicarbonate, it is determined that the ammonium bicarbonate dissolution temperature is most suitable at 20°C. The dissolution tank temperature is controlled at 20°C, plus or minus 1°C, and then ammonium bicarbonate is dissolved. Water is heated to 95°C. The experimental working medium was boosted by the circulating pump. Particularly, the data acquisition and analysis system started to record the temperature data when the flow passed through the heat transfer part. To obtain accurate data, each group of experiments was repeated three times.

The theoretical basis is the heat transfer basic equation based on Newton's cooling law and heat balance relationship.

The heat transfer basic equation is as follows:

4.1 $$Q=\alpha \cdot A\cdot \Delta t_{m}.$$

The logarithmic temperature is calculated as follows:

4.2 $$\Delta t_{m}=\frac{\Delta t_{\max}-\Delta t_{\min}}{\text{ln}(\Delta t_{\max})/(\Delta t_{\min})}.$$

Heat transfer:

4.3 $$Q=q_{V}\rho c_{P}(t_{c2}-t_{c1}).$$

Heat transfer coefficient expression:

4.4 $$\alpha =\frac{Q}{A\cdot \Delta t_{m}}.$$

By experiment *Q, A* and $\Delta t_{m}$, *α* can be obtained. The experimental device diagram is shown in figure 2.

The uncertainty analysis of the experiment is made based on the root-sum-square method. The temperature of the hot water and the solution are measured by K-type thermocouples, and the output signals are collected by the ADAM4118 temperature measurement module. Their errors are 0.75% and 0.1%, respectively, so the uncertainty is

4.5 $$\frac{\delta t}{t}=\sqrt{{\left(0.75\%\right)}^{2}+{\left(0.1\%\right)}^{2}}=0.757\%.$$

The velocity of the hot water and the solution are measured by the Pitot tube, and the uncertainty of the velocity is

4.6 $$\frac{\delta u}{u}=0.5\%.$$

The density of the saturated solution of ammonium bicarbonate at 20°C is 1.012 g cm^−3^, and $C_{P}=4.2\times 10^{3}\;\text{J}\;{\left(\text{kg}{\;}^{\circ }\text{C}\right)}^{-1}$. The experimental data are presented in table 4:

**Table 4.** Results of experiment at the initial working condition.

value	$t_{c1}$	$t_{c2}$	$T_{b1}$	$T_{b2}$	$\Delta t_{\max}$	$\Delta t_{\min}$	$\Delta t_{m}$	*Q*	*α*
1	21.6	22.2	80.2	73.2	58.0	51.6	54.75	2014.7	1071
2	21.6	22.2	78.3	71.6	56.1	50.0	52.99	2014.7	1010
3	21.6	22.1	76.2	69.4	54.1	47.8	50.88	1678.9	948
4	19.6	20.2	82.6	74.5	62.4	54.9	58.57	2065.7	1023
5	19.6	20.2	80.3	71.1	60.0	52.1	55.96	2065.7	1075
6	19.6	20.2	80.1	71.0	59.8	51.4	55.49	2065.7	1085

So the average heat transfer coefficient is $\alpha \text{ = 1052}\;\text{W}\;{\text{m}}^{-2}{\;}^{\circ }\text{C}$.

**Figure 3.**
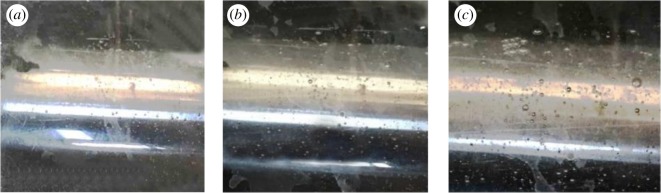
The experimental process phenomenon. (*a*) The beginning of the reaction. (*b*) The reaction has started for a period of time. (*c*) The fully carried out of the reaction.

Figure 3 shows the phenomenon of the experimental process. Bubbles are produced by heating ammonia bicarbonate solution. Figure 3*a* shows the beginning of the reaction, when the liquid has a small amount of bubbles, and the bubble diameter is small. Figure 3*b* shows the reaction for a period of time; then the number of bubbles has increased, and the bubble radius has increased. Figure 3*c* shows that the reaction has reached its vigorous extent, and the number and volume of bubbles have increased. The big bubbles can be seen clearly in this figure.

### 4.2. Heat transfer simulation by FLUENT

In the present study, the commercial computational fluid dynamics (CFD) package FLUENT 6.2 was used to model combustion and heat transfer in the thermal decomposition chamber. Using this software, two different models of the tube bundle are established, which are the row type and the fork type. Comparison of the two different models is done with regard to the heat transfer, length and number of heat transfer tubes, and the diameter of the outer tube is consistent. The transport equations for the standard k--ε model have been selected. The standard k--ε model is a model based on model transport equations for the turbulence kinetic energy (k) and its dissipation rate (ε).The modelling parameters are given in table 5.

**Table 5.** Modelling parameters.

	shell length	tube length	number of internal tubes	shell radius	inner tube radius	two inner tube spacing
row and fork type	5	5	19	0.8	0.1	0.3

**Figure 4.**
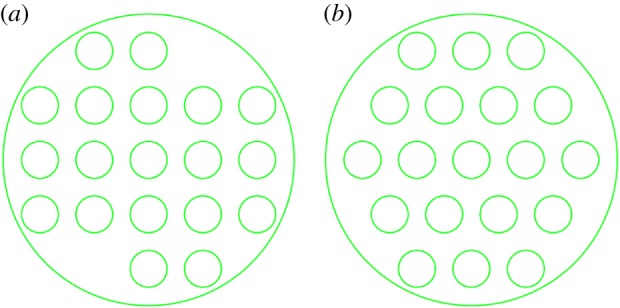
(*a*) Row-type side view. (*b*) Fork-type side view.

Figure 4*a,b* show the arranged 19 inner tubes, the inner tube diameter and relative position of the same.

After the Boolean operation, the shell and the tube are distinguished as two different fluid regions. In the subsequent operation, the inner tube area serves as a separate fluid area, and the outer portion of the housing of the inner tube serves as another fluid region. Boundary condition setting and grid division have been done. In the two types of arrangement, five boundary conditions are set, including cold-in, cold-out, hot-in, hot-out and heat-exchange wall.

**Figure 5.**
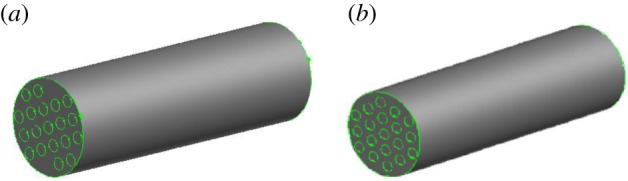
(*a*) The row-type after Boolean operation. (*b*) The fork-type after Boolean operation.

After obtaining the boundary condition and dividing the model of the grid, the resulting grid file can be imported into FLUENT, and the heat transfer coefficient is obtained by the experimental data, including the hot and cold fluid inlet temperature, the volume flow through the conduct of the heat transfer simulation experiment (figure 5).

**Figure 6.**
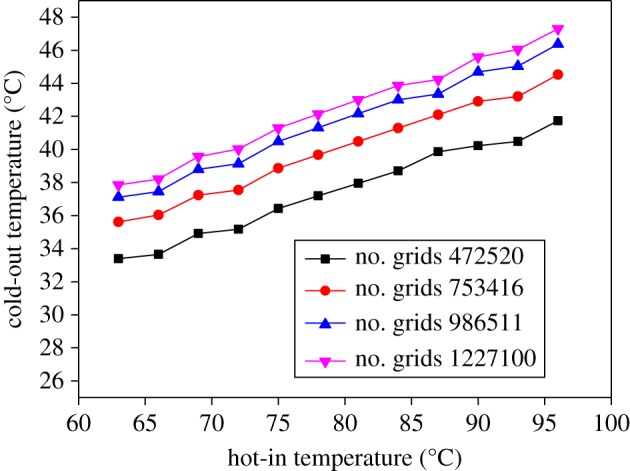
Temperature variation of cold-out with hot-in temperature change in different grids.

A grid independence study is carried out. It can be seen from figure 6 that when the number of grids reaches 986511, the cold outlet temperature changes little at the same hot inlet temperature as the number of grids increases, satisfying the accuracy requirements of the simulation results. To improve the efficiency of the calculation, we take the number of grids as 986 511 and start the subsequent calculation process.

**Figure 7.**
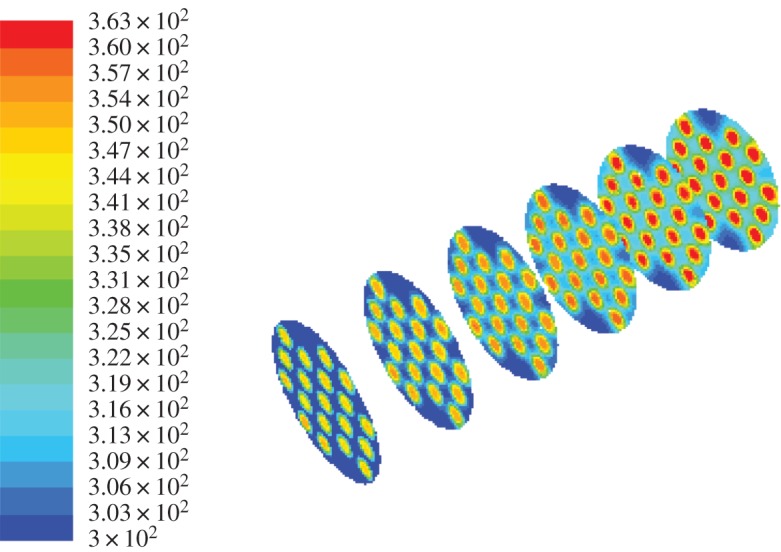
The diagram of row-type flow heat transfer simulation.

**Figure 8.**
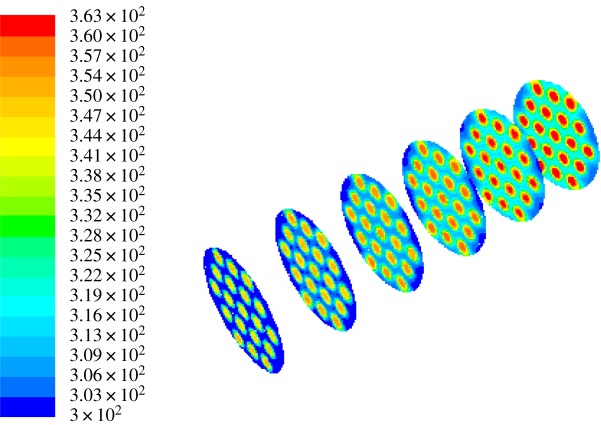
The diagram of fork-type flow heat transfer simulation.

It can be seen from figures 7 and 8 that the heat exchange occurs when the hot and cold fluids are subjected to convection. With it being closer to the inner tube part, the heat transfer effect is getting better. The heat transfer effect achieves the best in the centre of the casing. Contrasting the two figures, the heat transfer of the fork type is better than that of the row type. The cold outlet temperature of the row type is 316.03 K and that of the fork type is 317.28 K. It can be said that the flow heat transfer of the fork-type structure is better than the row-type flow heat transfer. So the fork-type structure is selected for the thermal decomposition chamber heat transfer structure.

**Figure 9.**
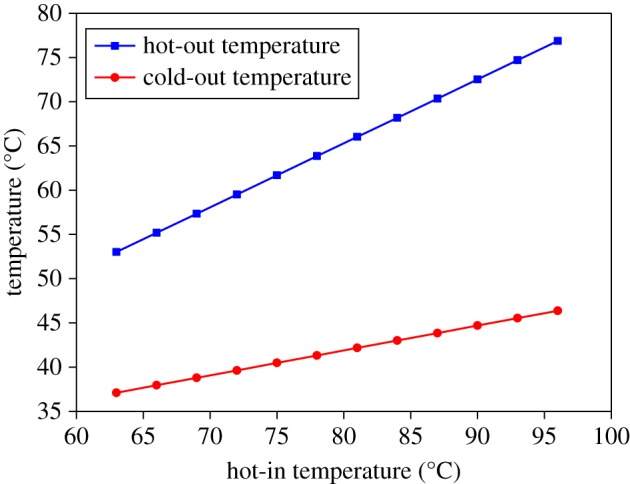
Temperature variation of cold-out and hot-out with hot-in temperature change.

**Figure 10.**
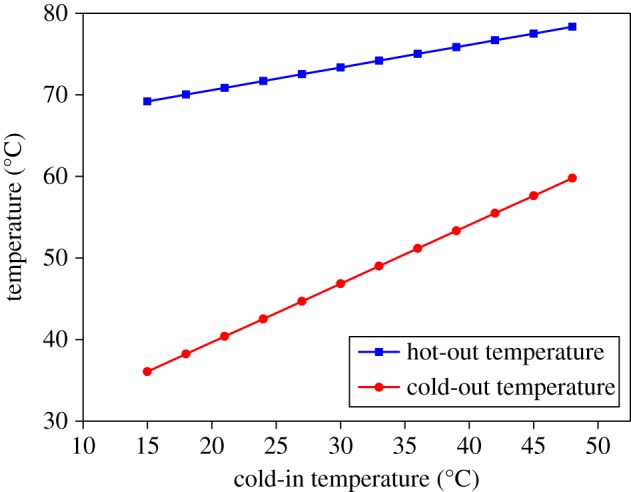
Temperature variation of cold-out and hot-out with cold-in temperature change.

Figure 9 is the case where the cold-in and hot-in flow rates remain constant and the cold-in temperature remains constant. By changing the temperature of the hot-in, the temperature of the hot-out and the cold-out is calculated separately. It can be seen that as the hot-in temperature increases, both the hot-out temperature and the cold-out temperature rise, and the hot-out temperature rises faster than the cold-out temperature. Similarly, figure 10 is the case where the cold-in and hot-in flow rates remain constant and the hot-in temperature remains constant. By changing the temperature of the cold-in, the temperature of the hot-out and the cold-out is calculated separately. It can be seen that as the cold-in temperature increases, both the hot-out temperature and the cold-out temperature rise, and the hot-out temperature rises slower than the cold-out temperature.

### 4.3. Simulation of ammonium bicarbonate thermal decomposition products by ASPEN

**Figure 11.**
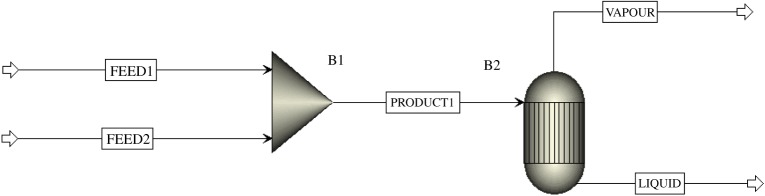
The process of ammonium bicarbonate decomposition simulated by ASPEN PLUS. FEED1, ammonium bicarbonate supply; FEED2, water supply; B1, mixer; PRODUCT1, ammonium bicarbonate solution; B2, reactor; VAPOUR, reaction gas outlet; LIQUID, reaction waste liquid outlet.

The amount of decomposition products of ammonium bicarbonate was simulated by the ASPEN software. The type of reactor used is Rstoic (stoichiometric reactor); furthermore, the thermodynamic properties and vapour liquid equilibrium were calculated by material balance. ASPEN simulated the ammonium bicarbonate solution decomposition reactor process, as shown in figure 11. FEED1 solid ammonium bicarbonate is mixed with FEED2 liquid water in mixer B1 to produce PRODUCT1 (ammonium bicarbonate solution). Ammonium bicarbonate solution is pumped into the reactor B2, and thermal decomposition takes place. Gas products are discharged through the VAPOUR mouth, and waste liquid through the LIQUID mouth.

The molar flow rate of ammonium bicarbonate is 0.17 mol s^−1^ and the flow rate is 2.78 mol s^−1^.

Initial process parameters:

(1) FEED1 ammonium bicarbonate molar flow rate: 0.17 mol s^−1^;
(2) FEED2 water molar flow: 2.78 mol s^−1^;
(3) Mixer B1 and reactor B2 Pressure: 0.101 MPa;
(4) Mixer B1 temperature: 30°C;
(5) Reactor B2 temperature: 95°C.

The simulation results are given in table 6.

**Table 6.** Simulation results.

flow	FEED1	FEED2	PRODUCT1	VAPOUR	LIQUID
temperature (°C)	30	30	20.11	95	95
pressure (MPa)	0.101	0.101	0.101	0.101	0.101
molar flow (mol s^−1^)	0.17	2.78	3.15	1.68	1.56
mass flow (kg h^−1^)	48.382	180.297	228.679	123.302	105.377
NH_4_HCO_3_ (mol s^−1^)	0.17	0	0	0	0
H_2_O (mol s^−1^)	0	2.78	2.787	1.387	1.549
CO_2_ (mol s^−1^)	0	0	0.007	0.156	0
NH_3_ (mol s^−1^)	0	0	0.001	0.142	0.009

**Figure 12.**
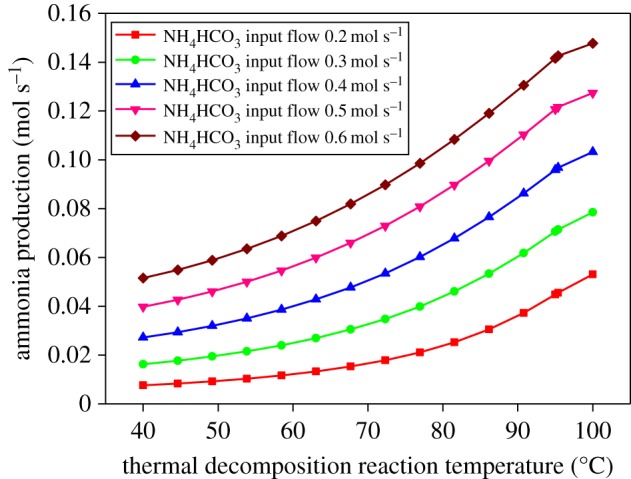
The change of ammonia volume at the outlet of thermal decomposition reactor with different initial ammonium bicarbonate input flow.

The change in the amount of NH_3_ at the gas outlet of the thermal decomposition reactor was observed by changing the initial feed rate of the bicarbonate and the reaction temperature of the thermal decompositions reactor. Figure 12 shows the change of ammonia volume at the outlet of the thermal decomposition reactor with different initial ammonium bicarbonate input flow. With the same ammonium bicarbonate input flow, but with higher thermal decomposition reaction temperature, the ammonia production is higher. Similarly, with the same thermal decomposition reaction temperature, but with greater ammonium bicarbonate input flow, the ammonia production is higher.

The heat transfer coefficient is obtained by a single-tube heat transfer coefficient determination experiment. The flow of the fork-type structure of heat transfer is better than the row-type flow heat transfer according to heat transfer simulation, so the fork-type structure is selected. Seen from the simulation of ammonium bicarbonate thermal decomposition products, the ammonia production is influenced by the thermal decomposition reaction temperature and the ammonium bicarbonate input flow.

## 5. Conclusion

In this article, an ammonia supply system for flue gas denitrification of low-speed marine diesel is proposed, and ammonium bicarbonate is selected as the denitrification agent. As the solubility of ammonia and carbon dioxide is different in water, the carbon dioxide will be separated and purified ammonia gas will be obtained. Through a heat transfer coefficient determination visualization experiment, the heat transfer coefficient *α* of the heat exchanger in the ammonia supply system is determined. The heat transfer coefficient is measured by experiment and the flow heat transfer simulation is conducted by FLUENT. Heat transfer simulation is carried out by using row-type and fork-type heat transfer structures. The thermal decomposition of ammonium bicarbonate is simulated by ASPEN PLUS. Based on the analysis made in this study, conclusions can be drawn as follows:

(1) The ammonia supply system for flue gas denitrification is proposed by using ammonium bicarbonate and waste heat from the diesel exhaust heat. Because of the difference in the solubility of ammonia and carbon dioxide in water, the carbon dioxide will be separated and purified ammonia gas will be obtained.
(2) The experiment for the single-tube heat transfer coefficient has been performed. The heat transfer coefficient is $\alpha =\text{1052}\;\text{W}\;{\text{m}}^{-2}{\;}^{\circ }\text{C}$ obtained by experiment. Moreover, uncertainty analysis of the experimental data has been done.
(3) Two different models of the tube, the row type and the fork type, have been simulated by FLUENT. The fork-type structure can be found better than the row type of flow heat transfer. Therefore, the fork-type structure is selected for design of the ammonium bicarbonate thermal decomposition chamber.
(4) The thermal decomposition of ammonium bicarbonate was simulated by ASPEN PLUS. The higher the thermal decomposition reaction temperature, the larger is the amount of ammonia production on the condition of the same input of ammonium bicarbonate solution. Similarly, the greater the input of ammonium bicarbonate solution, the larger is the amount of ammonia production, on the condition of the same thermal decomposition reaction temperature.

## Data accessibility

All experimental data required to repeat the analysis in this article are included within the article.
